# Editorial: Infectious diseases in maternal-fetal interface: role of immune system to combat this pathology and avoid or minimize a possible vertical transmission

**DOI:** 10.3389/fimmu.2026.1985460

**Published:** 2026-09-21

**Authors:** Bellisa Freitas Barbosa, Lamba Omar Sangaré, Ulrike Kemmerling

**Affiliations:** 1 Laboratory of Immunophysiology of Reproduction, Institute of Biomedical Science, Federal University of Uberlândia, Uberlândia, MG, Brazil; 2 Department of Biology, Texas A&M University, College Station, TX, United States; 3 Institute of Biomedical Science, School of Medicine, University of Chile, Santiago, Chile

**Keywords:** immune response, infectious disease, maternal-fetal interface, pathology, vertical transmission

The human maternal-fetal interface, represented primarily by the placenta, constitutes the main barrier between the maternal and fetal compartments during pregnancy. It consists of a maternal component, the decidua, and a fetal component formed by cells that give rise to the chorionic villi (1). The placenta originates from the trophoblast surrounding the blastocyst and, following implantation, these cells differentiate into villous and extravillous cytotrophoblasts (2). Villous cytotrophoblasts give rise to the syncytiotrophoblast, which covers the placental villi, acts as a barrier against pathogens, and participates in maternal-fetal exchange (3, 4). Extravillous cytotrophoblasts, in turn, contribute to spiral artery remodeling and establish a tolerogenic environment through interactions with decidual immune cells (5–7). Together with the basal lamina, mesenchyme, and fetal blood vessels, these structures form the placental barrier (4).

The integrity of this barrier is essential to protect the fetus from pathogens (4, 8, 9). However, some pathogens, such as protozoa and viruses, can cross the placental barrier and impair fetal development (10). This challenge is further complicated by the maternal immune system’s need to adapt throughout pregnancy. During the first trimester, a Th1 response characterized by IL-8, IFN-γ and TNF-α contributes to implantation, vascular remodeling and uterine repair, but its persistence may promote pregnancy loss (11, 12). During the second trimester, a Th2 response predominates, characterized by IL-4, TGF-β1 and IL-10, supporting fetal development and maternal tolerance to the allogeneic fetus. Toward the end of pregnancy, the Th1 response becomes predominant again, contributing to preparation for labor (13–15). An imbalance between these responses may contribute to complications such as preeclampsia (16, 17), whereas a Th2-skewed environment may increase susceptibility to infections and facilitate vertical transmission (9, 18). Thus, infections at the maternal-fetal interface represent a unique immunological challenge, requiring a balance between pathogen control and the immune tolerance necessary to sustain pregnancy.

The primary objective of this Research Topic was to bring together studies addressing the interactions among pathogens, the placenta and the immune system, with emphasis on the mechanisms underlying placental invasion, immune responses and vertical transmission, as well as diagnostic, therapeutic and preventive strategies. Eight articles were published, including original research studies, reviews and a brief research report, addressing different aspects of infections during pregnancy and their consequences.

Mbianda et al. investigated γδ T-cell differentiation in placental malaria and demonstrated compartment-specific differentiation, with an increased TEMRA phenotype associated with IL-8 and IL-33, as well as enhanced mitochondrial metabolic activity. These findings suggest that cytokines and metabolic reprogramming modulate γδ T-cell function and may represent potential therapeutic targets.

Mizobuchi et al. evaluated the effects of maternal *Leishmania donovani* infection on offspring immune responses. Although the offspring did not develop persistent infection or acquired immune memory, they showed sex-specific immunopathological alterations: males had increased erythrophagocytosis and anemia, whereas females had increased parasitism and hepatic inflammation. These findings demonstrate that maternal infection can induce long-lasting alterations in offspring immunity.

Choi et al. investigated a vaccine strategy against different strains of congenital cytomegalovirus (cCMV). The combination of antibodies targeting glycoprotein B (gB) and a T-cell response against IE1 antigen reduced CMV transmission to undetectable levels, whereas the gB-only vaccine provided limited protection. These results highlight the potential of combining humoral and cellular immune responses to achieve cross-strain protection against cCMV.

Jiménez-Escutia et al. evaluated how gestational diabetes-associated hyperglycemia affects the placental response to *Streptococcus agalactiae* (GBS). The combination of hyperglycemia and infection increased bacterial proliferation and disrupted the syncytial barrier, while reducing TLR-2 and inflammatory mediators such as TNF-α, MCP-1, and MIP-1β, thereby impairing the recruitment of monocytes and NK cells. These findings suggest that hyperglycemia promotes bacterial persistence and loss of placental barrier integrity.

Kahraman and Celina reviewed 38 cases of Crimean-Congo hemorrhagic fever during pregnancy, reporting maternal survival of 68% and fetal or neonatal survival of only 39%. The interaction between pregnancy’s Th2-predominant immune environment and endothelial and hepatic alterations may promote viral replication, hemorrhage, and coagulopathy, while also mimicking conditions such as HELLP syndrome and severe preeclampsia. The authors emphasize the need for early diagnosis, virological confirmation, and safe antiviral therapies.

SARS-CoV-2 also represents an important threat during pregnancy and has been associated with adverse outcomes, including maternal mortality, preterm birth, and stillbirth (19), with the virus also detected in the placenta and fetus (20, 21). In this context, Packer et al. demonstrated that vaccination was associated with a lower incidence of severe maternal disease, despite higher levels of some cytokines, including TNF-α, suggesting immune priming. They observed no specific fetal inflammatory response, supporting a role for vaccination in modulating the maternal immune response.

Yuan et al. evaluated the neutralizing antibody response to the SARS-CoV-2 NB.1 variant in domestic cats. NB.1 exhibited greater immune escape than JN.1, with a 2.9-fold reduction in neutralizing antibody titers, although none of the animals tested showed evidence of infection. Although the study did not directly investigate the maternal-fetal interface, it contributes to understanding immune escape and variant surveillance, both of which are relevant to developing vaccination strategies.

Finally, Chikungunya virus (CHIKV) can cause significant morbidity in congenitally infected children (22), and viral antigens have been detected in different placental cell types, accompanied by alterations in immune cells and cytokines (23). In this context, Zeng et al. reviewed the mechanisms underlying chronic chikungunya-associated arthritis, proposing a cascade involving viral persistence, myeloid cell metabolic dysregulation, Treg/Th17 imbalance, and autoimmune-like tissue injury. Although not focused on congenital infection, this study expands our understanding of viral persistence and immunometabolic dysregulation during arboviral infections, highlighting potential intervention targets in maternal infection.

Collectively, the eight articles highlight the complexity of the interactions among pathogens, metabolism, immune responses, and the placental barrier. These studies reinforce that understanding the mechanisms governing pathogen persistence, control, and transmission is essential for developing more effective diagnostic, preventive, and therapeutic strategies, ultimately contributing to reducing the impact of congenital infections on maternal and fetal health.

## References

[1] Knofler M Haider S Saleh L Pollheimer J Gamage TKJB James J . Human placenta and trophoblast development: key molecular mechanisms and model systems. Cell Mol Life Sci. (2019) 76:3479–96. doi: 10.1007/s00018-019-03104-6 31049600 PMC6697717

[2] Aplin JD Myers JE Timms K Westwood M . Tracking placental development in health and disease. Nat Rev Endocrinol. (2020) 16:479–940. doi: 10.1038/s41574-020-0372-6 32601352

[3] Aplin JD . Developmental cell biology of human villous trophoblast: current research problems. Int J Dev Biol. (2010) 54:323–29. doi: 10.1387/ijdb.082759ja 19876840

[4] Ander SE Diamond MS Coyne CB . Immune responses at the maternal-fetal interface. Sci Immunol. (2019) 4:1–11. doi: 10.1126/sciimmunol.aat6114 30635356 PMC6744611

[5] Burton GJ Jauniaux E . The cytotrophoblastic shell and complications of pregnancy. Placenta. (2017) 60:134–39. doi: 10.1016/j.placenta.2017.06.007 28651899

[6] Polheimer J Vondra S Baltayeva J Beristain AG Knofler M . Regulation of placental extravillous trophoblasts by the maternal uterine environment. Front Immunol. (2018) 9:2597. doi: 10.3389/fimmu.2018.02597 30483261 PMC6243063

[7] Turco MY Moffett A . Development of the human placenta. Development. (2019) 146:22. doi: 10.1242/dev.163428 31776138

[8] Arora N Sadovsky Y Dermody TS Coyne CB . Microbial vertical transmission during human pregnancy. Cell Host Microbe. (2017) 21:561–67. doi: 10.1016/j.chom.2017.04.007 28494237 PMC6148370

[9] Costa ML Nobrega GM Antolini-Tavares A . Key infections in the placenta. Obstet Gynecol Clin North Am. (2019) 47:133–46. doi: 10.1016/j.ogc.2019.10.003 32008664

[10] Ostrander B Bale JF . Congenital and perinatal infections. Handb Clin Neurol. (2019) 162:133–53. doi: 10.1016/B978-0-444-64029-1.00006-0 31324308

[11] Wang W Sung N Gilman-Sachs A Kwak-Kim J . T helper (Th) cell profiles in pregnancy and recurrent pregnancy losses: th1/th2/th9/th17/th22/tfh cells. Front Immunol. (2020) 11:2025. doi: 10.3389/fimmu.2020.02025 32973809 PMC7461801

[12] Sykes L Macintyre DA Yap XJ Teoh TG Bennett PR . The Th1:th2 dichotomy of pregnancy and preterm labour. Mediators Inflammation. (2012) 2012:967629. doi: 10.1155/2012/967629 22719180 PMC3376783

[13] Roth I Corry DB Locksley RM Abrams JS Litton MJ Fisher SJ . Human placental cytotrophoblasts produce the immunosuppressive cytokine interleukin 10. J Exp Med. (1996) 184:539–48. doi: 10.1084/jem.184.2.539 8760807 PMC2192727

[14] Rezende-Oliveira K Silva NM Mineo JR Rodrigues Junior V . Cytokines and chemokines production by mononuclear cells from parturient women after stimulation with live *Toxoplasma gondii*. Placenta. (2012) 33:682–87. doi: 10.1016/j.placenta.2012.05.013 22742727

[15] Keelan JA Marvin KW Sato TA Coleman M Mccowan LM Mitchell MD . Cytokine abundance in placental tissues: evidence of inflammatory activation in gestational membranes with term and preterm parturition. Am J Obstet Gynecol. (1999) 181:1530–36. doi: 10.1016/s0002-9378(99)70400-x 10.1016/s0002-9378(99)70400-x 10601939

[16] Saito S Sakai M . Th1/Th2 balance in preeclampsia. J Reprod Immunol. (2003) 59:161–73. doi: 10.1016/S0165-0378(03)00045-7 12896820

[17] Colucci F . The immunological code of pregnancy. Science. (2019) 365:862–63. doi: 10.1126/science.aaw1300 31467207

[18] Jamieson DJ Theiler RN Rasmussen SA . Emerging infections and pregnancy. Emerg Infect Dis. (2006) 12:1638–43. doi: 10.3201/eid1211.060152 17283611 PMC3372330

[19] Li A Schwartz DA Vo A Vanabel R Coler C Li E . Impact of SARS-CoV-2 infection during pregnancy on the placenta and fetus. Semin Perinatol. (2024) 48:151919. doi: 10.1016/j.semperi.2024.151919 38897829 PMC11288977

[20] Rakheja D Treat K Timmons CF Carrillo D Miller SE Stroberg E . SARS-CoV-2 immunohistochemistry in placenta. Int J Surg Pathol. (2022) 30:393–96. doi: 10.1177/10668969211067754 34939436 PMC9111943

[21] Reagan-Steiner S Bhatnagar J Martines RB Milligan NS Gisondo C Williams FB . Detection of SARS-CoV-2 in neonatal autopsy tissues and placenta. Emerg Infect Dis. (2022) 28:510–17. doi: 10.3201/eid2803.211735 35138244 PMC8888232

[22] Evans-Gilbert T . Vertically transmitted chikungunya, Zika and dengue virus infections: The pathogenesis from mother to fetus and the implications of co-infections and vaccine development. Int J Pediatr Adolesc Med. (2020) 7:107–11. doi: 10.1016/j.ijpam.2019.05.004 33094137 PMC7567994

[23] Salomão N Rabelo K Avvad-Portari E Basílio-De-Oliveira C Basílio-De-Oliveira R Ferreira F . Histopathological and immunological characteristics of placentas infected with chikungunya vírus. Front Microbiol. (2022) 17:1055536. doi: 10.3389/fmicb.2022.1055536 36466642 PMC9714605

